# Lingual alveolar soft part sarcoma with absent TFE3 rearrangement

**DOI:** 10.1002/cnr2.1298

**Published:** 2020-10-07

**Authors:** Saeed Alshahrani, Abdullatif Khan

**Affiliations:** ^1^ College of Medicine King Saud bin Abdulaziz University for Health Sciences Riyadh Saudi Arabia; ^2^ Department of Pathology and Laboratory Medicine King Abdulaziz Medical City Riyadh Saudi Arabia

**Keywords:** glossectomy, lingual cancer, oral, sarcoma, tumor

## Abstract

**Background:**

Lingual ASPS is extremely rare and aggressive tumor. rearrangement is typically detected in ASPS patients using FISH analysis.

**Aim:**

To present the clinical, histopathological, and radiological features of lingual Alveolar Soft Part Sarcoma.

**Method:**

A 30‐year‐old male presented with a painless, slowly growing mass of the tongue. Initial impression was of benign vascular lesion. Later, the patient became symptomatic as the mass progressed in size, which necessitated further investigations.

**Result:**

A lip‐split, mandibulotomy was performed for the excision of the tumor and revealed an alveolar soft part sarcoma with PAS‐positive, diastase resistant intracytoplasmic granules. However, molecular analysis using FISH was negative for TFE3 rearrangement. Patient underwent partial glossectomy with postoperative radiotherapy.

**Conclusion:**

Clinical and pathological correlation of ASPS is very useful to reach a proper diagnosis.

## 1 INTRODUCTION

Alveolar soft part sarcoma (ASPS) is a very rare and aggressive malignant neoplasm of uncertain histogenesis.^1^ It comprises less than 1% of all soft tissue sarcomas, most commonly arising in lower extremities. Head and neck neoplasms occur in 27% of ASPS cases, and 25% of those cases arise in the tongue. Typically, ASPS of the tongue presents with a painless, slowly growing mass, and is more likely to occur in females between 15 and 35 years old.^2^ Here, we describe the clinical, radiological, and histological features of alveolar soft part sarcoma (ASPS) of the tongue.

## 2 CASE

A 30‐year‐old male presented with a painless swelling on the left base of the tongue surface, first noted 3 months back. The mass was slowly progressing in size. There were no other associated symptoms. Clinical examination revealed a nontender swelling on the left posterior part of tongue. The mass was firm in consistency, oval in shape, and had an intact mucosal covering, not associated with ulceration, and was not attached to any underlying tissue. Neck examination revealed palpable ipsilateral submandibular and jugulodigastric lymph nodes. A clinical diagnosis of hemangioma was suspected. CT angiogram revealed a left sided highly vascular lesion. Three months later, the patient came back with symptomatic dysphagia, chewing difficulty, and difficulty in breathing especially while sleeping, and CT scan showed a slight increase in the size of the mass.

Since the possibility of tongue cancer could not be ruled out in the presence of alarming compressive symptoms, incisional biopsy was planned. During the procedure, a significant bleeding was encountered which was difficult to be controlled with diathermy, local anesthesia, pressure packing and surgicel. Thus, it was decided to abort the procedure after hemostasis was completely secured, and the patient was extubated without any complications. Following incisional biopsy, selective embolization of the left lingual artery achieved a near complete devascularization of the mass. Then the patient was sent back for lip‐split, mandibulotomy for access and excision of the left tongue tumor. Suturing on the muscle and in the mucosa was performed with 2‐0 Vicryl and 3‐0 Vicryl and the defect was closed primarily. Postoperative period was unremarkable.

Three weeks after excision of the tumor, radiological evaluation was performed using PET/Scan and neck MRI. PET/Scan showed a moderate hypermetabolism at the site of surgical site and at the level of 2A lymph nodes with no evidence of distant metastasis. Neck MRI showed postsurgical changes of the tongue with enhancing left upper cervical lymphadenopathy.

**FIGURE 1:**
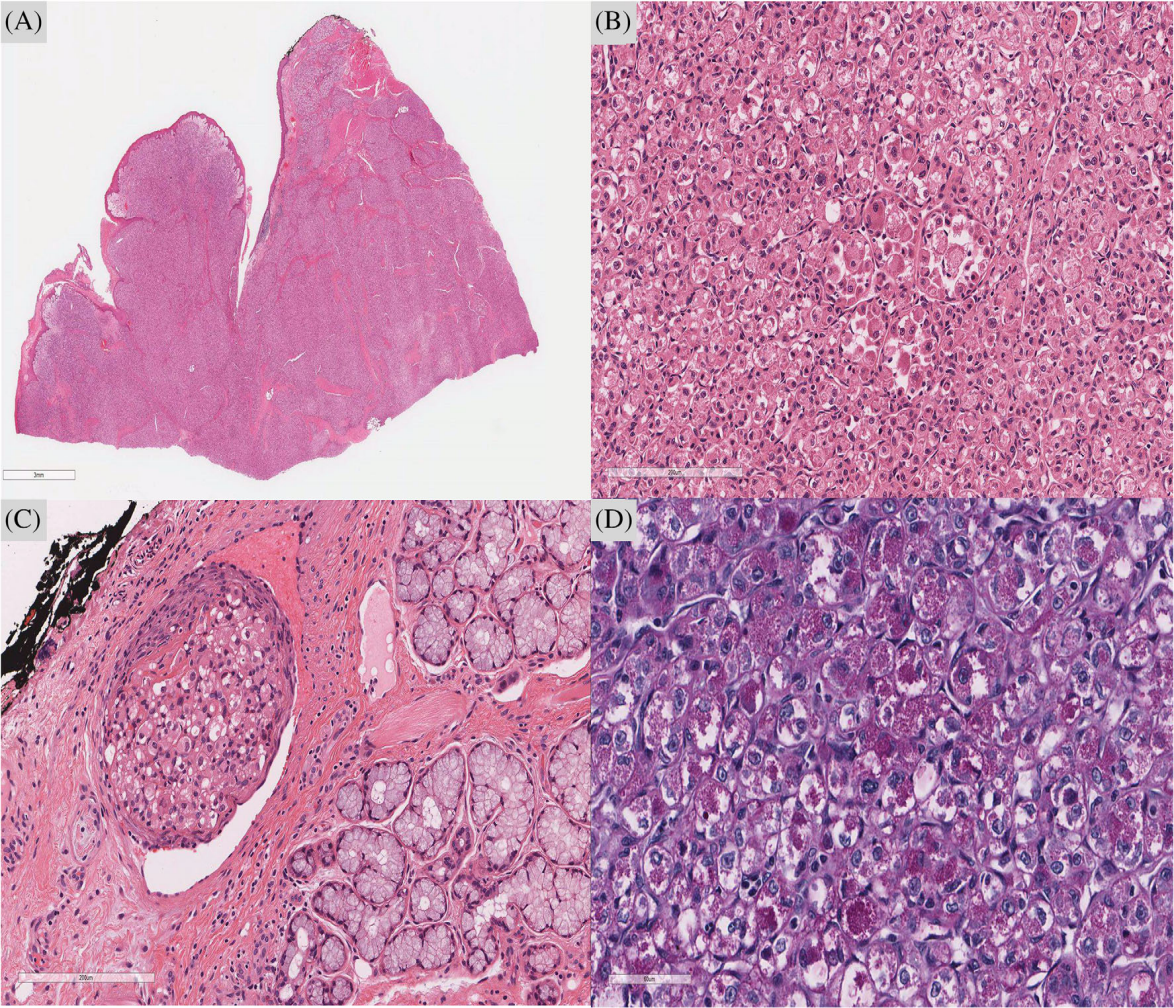
ASPS. A, Lingual tissue extensively replaced by the tumor. B, High power image shows large cells with distinct borders, and clear to lightly esinophilic cytoplasm with prominent nucleoli arranged in a pseudoalveolar pattern. C, Vascular invasion. D, Periodic acid–Schiff (PAS) staining is shows PAS positive diastase resistant intracytoplasmic inclusions in some cells of the tumor.**A,B,C: Hematoxylin and eosin (H&E) stains. D: PAS‐D stain

Following the excision of the tumor, the specimen was sent to the Anatomic Pathology Department. Grossly, it was a cauliflower‐like ulcerating mass lesion which was 4.8 cm in greatest dimension. Histopathological examination revealed a malignant neoplasm formed of large, polygonal cells with clear to lightly esinophilic cytoplasm and hyperchromatic nuclei arranged in a pseudoalveolar pattern with prominent capillary vasculature. Perineural invasion and extensive lymphovascular invasion were present. PAS‐positive, diastase resistant intracytoplasmic granules were present (Figure 1).

**FIGURE 2:**
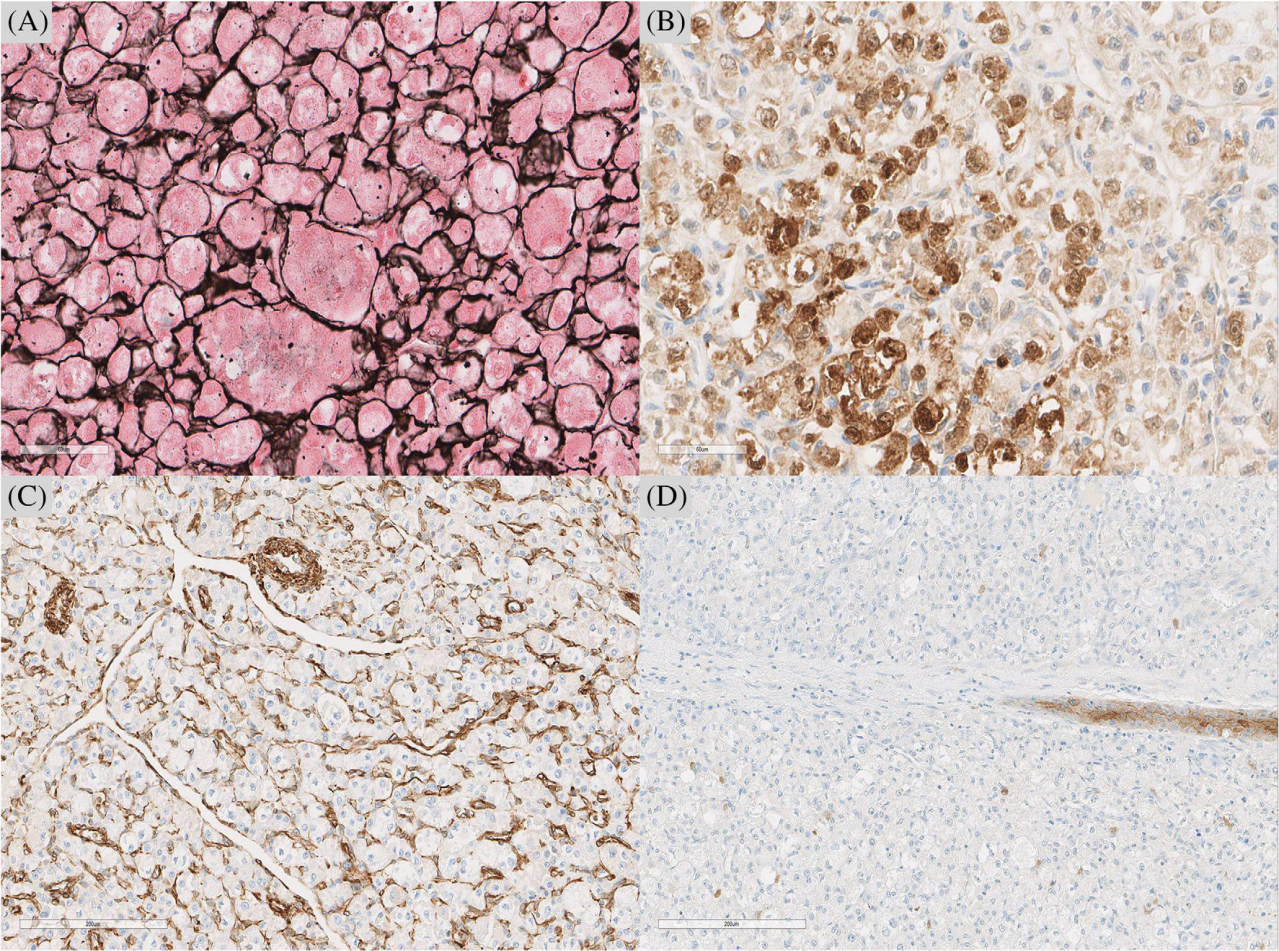
immunohistochemical reactivity and special staining. A, Reticulin stain highlighted the nested pattern. B, immunohistochemistry showed positive staining for neuron specific enolase (NSE). C, Vimentin. D, No stain for Cytokeratin

Immunohistochemical studies ruled out mimickers (Figure 2). Tumor cells were negative for Desmin, SMA, S100, CD34, Myo‐D1, Myogenin, PAN CK, Synaptophysin, Chromogranin, CD56, Melan A, and HMB45. A diagnosis of primary lingual Alveolar Soft Part Sarcoma was confirmed. Molecular studies were performed. Interestingly, FISH analysis molecular analysis was negative for TFE3 rearrangement. Therefore, a variant TFE3 fusion type or a fusion involving a related gene such as TFEB were suspected.

As the histopathology report showed involved surgical margin, patient agreed to have further resection of the lesion through partial glossectomy. Further excision revealed multiple residual foci of ASPS and a close deep resection margin. Left hemiglossectomy plus reconstruction of the left side of the tongue and the floor of the mouth with radial forearm free flap were performed. Adjuvant radiotherapy was completed 4 months postoperatively. A follow up period of 1 year was unremarkable except for multiple bilateral lung micronodules that remained unchanged on successive evaluation with chest imaging. Patient was stable and fully able to perform daily activities without restriction.

## 3 DISCUSSION

Alveolar soft part sarcoma (ASPS) mainly affects young adults and comprises less than 1% of all soft tissue sarcomas.^2^ To date, there have been 44 reported cases of ASPS in the oral cavity, and only 11 of which occurred in the base of the tongue.^3^ ASPS of tongue presents as a painless, well defined, slowly enlarging mass. Patients may be asymptomatic initially, but as the mass gets bigger, it may cause dysphagia, dysphonia or mild discomfort. Pulsation with a thrill is often felt on clinical examination.^4^ Extensive vascular invasion is typical in ASPS and commonly presents with metastasis at diagnosis. Most common site of metastasis is lung, followed by brain and bone. It involves lymph nodes.^5^

It is still controversial whether ASPS is of neuroendocrine or myogenic origin. Initially, ASPS was thought to be a malignant variant of granular cell myoblastoma which comes from a neuroendocrine origin. However, recent immunohistochemical studies have proven the absence of neuroendocrine differentiation.^2^

**FIGURE 3:**
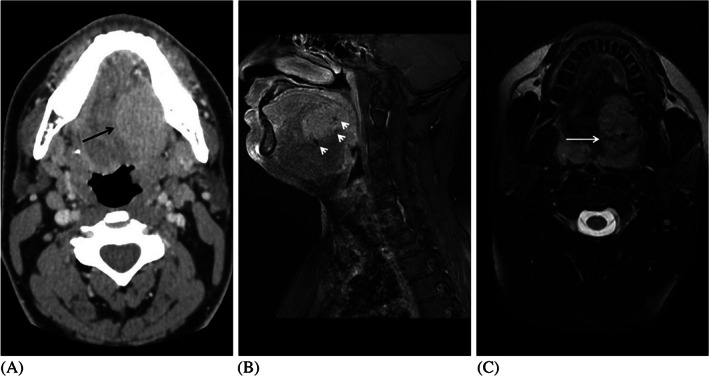
imaging findings of ASPS. A, Axial contrast enhanced computed tomography demonstrates a homogenous well defined hypervascular lesion in left posterior tongue (white arrow). B, Sagittal enhanced T1 weighted image demonstrates a tongue mass with homogenous pattern of contrast en‐hancement with flow void structures (white arrows). C, Axial unenhanced T2 weighted image demonstrates a hyperintense tongue mass with flow voids (white long arrow) and areas of slightly higher intensities (black short arrows) due to tumor necrosis

ASPS could be confused with hemangioma since both look similar in term of clinical and radiological pattern. Unlike hemangioma, ASPS is not present at birth and the growth pattern of the lesion is typically slow. Hemangioma appears as a well‐defined hyperattenuated lesion in CT without contrast and appears homogeneously intense in CT with contrast^5^ (Figure 3). Enhanced T1 weighted image showed increased signal intensity of the mass with flow void structures denoting vascular lesion which further made our case likely to be hemangioma in the beginning (Figure 3). Also, both conditions have high signal intensity on T2‐weighted images which makes it difficult to differentiate hemangioma from ASPS with imaging alone (Figure 3).

Histologically, ASPS often looks benign in a deceptive manner. Cells are typically of uniform size and shape with eosinophilic cytoplasm and prominent nucleolus without atypical mitosis or nuclear pleomorphism. ASPS characteristically appears in a pseudoalveolar pattern as alveolar cells get necrotic centrally and lose their cohesiveness (Figure 1).^2^ Positive Periodic acid‐Schiff (PAS), diastase resistant, intracytoplasmic granules are a pathognomonic histologic feature of ASPS (Figure 1). In addition to histopathological pattern, IHC studies are helpful to rule out differential diagnosis of ASPS such as granular cell tumor, paraganglioma, metastatic renal carcinoma (RCC) and alveolar rhabdomyosarcoma (ARMS).^6^

Recently, it was proposed that the unbalanced translocation, del (17) t(X,17) (p11,p25), which results in the formation of ASPL‐TFE3 transcript fusion detected on tumor cell, could specifically explain the tumorigenesis of ASPS.^7^ Immunohistochemical reactivity for TFE3 in ASPS usually stains weak to moderately positive.^7^ Sharain et al compared TFE3 rearrangement immunohistochemistry results between two laboratories.^8^ The overall sensitivity of TFE3 immunohistochemistry to detect TFE3‐rearranged neoplasms was 85% in laboratory A, and 70% in laboratory B. The difference in detection rate could be attributed to different fixation or processing techniques used by each laboratory, yet the presence or absence of immunohistochemical reactivity was not sufficient alone to confirm the diagnosis of ASPS since the specificities of IHC in lab A and lab B were 57% and 95% respectively. Also, a discordance between molecular and immunohistochemistry findings was reported in certain neoplasm in term of TFE3 rearrangement.^8^

Although FISH analysis remained extremely sensitive and specific to detect Xp11.23 translocation associated with ASPS and Renal cell carcinoma (RCC), the possibility of chromosomal abnormalities could not be ruled out by FISH analysis alone.^9^ In fact, a recent clinicalpathological review of 13 ASPS cases failed to show TFE3 rearrangements in four cases using FISH.^10^ Our case was negative for TFE3 rearrangement, but the correlated clinical and pathological features confirmed the diagnosis of primary Alveolar soft part sarcoma. Exploring the sensitivity of FISH testing to detect TFE3 fusion gene in ASPS cases could be difficult due to the rarity of reported cases and inability to identify the fusion partner of TFE3 at certain circumstances.^11^ Also, the possibility of unknown fusion variant could not be ruled out by molecular tests.^11^ Argani et al recently described a novel intrachromosomal Xp11.2 inversion (RBM10‐TFE3 gene fusion) occurred in RCC that initially presented with negative TFE3 break‐apart by FISH.^9^ Therefore, further studies are necessary to consolidate the molecular diagnostic capabilities to allow an accurate detection and confirmation of ASPS fusions genes.

Complete Surgical excision of the primary tumor is the treatment of choice to prevent local recurrence. Bleeding might be encountered, especially if the tongue lesion is located posteriorly.^2^ In our case, first surgical resection procedure was aborted due to the risk of massive bleeding. Preoperative embolization of the left lingual artery achieved near complete devascularization and made further surgical resection accessible. Although adjuvant therapy is still controversial, radiotherapy as used in our patient is recommended when surgical resection alone is inadequate, particularly in cases with remaining residual or metastatic lesions.^12^, ^13^

## 4 CONCLUSION

Lingual ASPS is an extremely rare tumor. To the best of our knowledge, this is the first case to report ASPS of the tongue in Saudi Arabia. Clinically and radiologically, ASPS can be misdiagnosed as hemangioma. The nature of tumor location and behavior made the diagnosis and management of this case more challenging. Therefore, careful evaluation of any hypervascular growing mass of the tongue should be done.

Histologic findings provide a reliable diagnostic method. Although molecular methods provide a great support to the diagnosis of ASPS, the absence of TFE3 fusion gene does not always rule out ASPS. Preoperative embolization for ASPS might be useful to avoid the risk of extensive bleeding during surgery.

## ETHICS STATEMENT

Informed consent was obtained to publish this report.

## CONFLICT OF INTEREST

The authors declared no potential conflicts of interest with respect to the research, authorship, and/or publication of this article.

## AUTHOR CONTRIBUTIONS

Saeed Alshahrani: wrote and edited the manuscript.

Abdullatif Khan: contributed to the writing and literature review.

## Data Availability

Some of the information in this case contains sensitive material and will not be disseminated to secure the patient’s confidentiality, but can be disclosed if necessary.
